# Effect of Nb concentration on the spin-orbit coupling strength in Nb-doped SrTiO_3_ epitaxial thin films

**DOI:** 10.1038/s41598-018-23967-2

**Published:** 2018-04-10

**Authors:** Seong Won Cho, Milim Lee, Sungmin Woo, Kanghoon Yim, Seungwu Han, Woo Seok Choi, Suyoun Lee

**Affiliations:** 1 Electronic Materials Research Center, Korea Institute of Science and Technology, Seoul, 02792 Korea; 2 Division of Nano & Information Technology, KIST School, Korea University of Science and Technology, Daejon, 34316 Korea; 3 Department of Physics, Sungkyunkwan University, Suwon, 16419 Korea; 4 Department of Materials Science and Engineering, Seoul National University, Seoul, 08826 Korea

## Abstract

Several oxide materials have attracted much interest for the application in spintronic devices due to unusual properties originating from the strongly correlated orbital and spin degrees of freedom. One missing part in *oxide spintronics* is a good spin channel featured by strong spin-orbit coupling (SOC) which enables an efficient control of the electron’s spin. We have systematically investigated the dependence of the SOC strength of Sr(Nb_*x*_Ti_1−*x*_)O_3_ thin films on Nb concentration (*n*_Nb_ = 2~20 at. %) as a deeper exploration of a recent finding of the strong SOC in a heavily Nb-doped SrTiO_3_ (Sr(Nb_0.2_Ti_0.8_)O_3_) epitaxial film. Apart from a finding of a proportionality of the SOC to *n*_Nb_, we have observed an intriguing temperature dependence of the SOC strength and the anisotropic magnetoresistance (MR) in the intermediate *n*_Nb_ region. These phenomena are associated with the temperature dependence of Landé g-factor and the change of the band structure, which is consistent with the result of density functional theory (DFT) calculation.

## Introduction

Electronic devices based on conventional semiconductors are facing the limit of evolution due to the huge consumption of energy, generation of excessive heat, monotonic functionality, and inextensible miniaturization. “Oxide-spintronics”, based on a broad spectrum of electrical and magneto-electrical properties originating from the interplay between orbital and spin degrees of freedom combined with the strong correlation nature of electrons in transition-metal oxides, is considered as one of the promising solutions^1–8^. For the development of high-performance spintronic devices using oxide materials, an essential ingredient is a good spin channel, where the electron’s spin can be manipulated at will within an infinitesimal energy and length scale.

Recently, observable effects of spin-orbit coupling (SOC) have been reported in various material systems, for example, topological insulators^9,10^, two-dimensional materials^11^, and some oxide materials such as LaAlO_3_/SrTiO_3_ (LAO/STO) heterostructure^12,13^ and pyrochlore iridates (*A*_2_Ir_2_O_7_, where *A* = yttrium and lanthanide element)^14–16^. In the former, a tunable and moderate strength SOC was reported while, in the latter, electrical and magnetic properties were reported to be dominated by the strong SOC. More recently, we found that a heavily Nb-doped SrTiO_3_ (SrNb_0.2_Ti_0.8_O_3_) epitaxial film grown on STO showed the strong SOC and the high carrier mobility – ideal characteristics for a good spin channel – which resulted in a large linear magnetoresistance (LMR)^17^. In this study, we have performed a systematic study on the magnetotransport properties of SrNb_*x*_Ti_1−*x*_O_3_ films with varying Nb concentration (*n*_Nb_) in the range of 2~20 at. % to find that the magnetotransport property is dominated by three-dimensional weak antilocalization (WAL)^18,19^ and that the SOC strength is proportional to *n*_Nb_. Furthermore, a few intriguing properties are also found, for example, non-monotonic temperature dependence of the SOC and anisotropy of MR in the intermediate *n*_Nb_ region.

Nb-concentration split SrTiO_3_ (Nb:STO) films were fabricated at 700 °C in 10^−5^ Torr of oxygen partial pressure using pulsed laser epitaxy (PLE). Laser (248 nm; IPEX 864, Lightmachinery, Nepean, Canada) fluence of 1.5 J/cm^2^ and repetition rate of 5 Hz was used. SrTiO_3_ and Sr(Nb_0.2_Ti_0.8_)O_3_ targets were used to systematically modify the Nb:STO concentration, by controlling the ablation ratio between the two targets within a unit cell thickness^20^. The advantage of using co-ablation of two targets instead of using Nb:STO solid solution target with different *n*_Nb_ was to avoid additional complexity and quality issues due to preparing distinctive targets. The thickness of the Nb:STO thin films was 16 ± 1 nm, as measured by X-ray reflectometry (XRR). The atomic structure and epitaxy relation of the thin films were characterized using high-resolution X-ray diffraction (XRD) (Rigaku, Smartlab) (in the Supplementary Information, Figure S1).

For the measurement of magnetotransport properties, a specimen was cut into 2(width) × 5(length) mm^2^ and six electrical contacts were formed by indium. After wiring, the sample was inserted in a commercial cryogen-free cryostat (CMag Vari-9, Cryomagnetics Inc.) and the resistance was measured using a Source-Measure Unit (Keithley 2612A) and a nano-voltmeter (Keithley 2182).

For DFT calculation, Vienna Ab initio Simulation Package (VASP)^21^ with PAW potentials. We employ HSE06 hybrid functional for the exchange-correlation functional and the energy cutoff of the plane-wave basis set to 450 eV for all calculation. For structural optimization, the 4 × 4 × 4 Monkhorst-pack **k**-point sampling is used for primitive cell of SrTiO_3_ and SrNbO_3_ in cubic perovskite structure. The calculated lattice parameter of SrTiO_3_ and SrNbO_3_ are 3.883 Å and 4.017 Å, respectively, which are consistent with experimental values^22,23^ with error less than 1%. We use the lattice parameter of 3.905 Å for the strained SrNbO_3_ that corresponds to the experimental lattice of SrTiO_3_. To calculate the density of states (DOS) of unstrained and strained SrNbO_3_, we use 6 × 6 × 6 Monkhorst-pack **k**-point sampling and tetrahedron method for Brillouin-zone integration. For the calculation of Nb:STO, we use the 130-atom supercell that one Ti atom is substituted by Nb which correspond to the doping concentration of 3.8 at %. For **k**-point sampling of defect cell, we use Γ-only k-point which corresponds to same **k**-point density compared to primitive cell. For DOS calculation of Nb:STO, we also use Γ-only **k**-point and Gaussian smearing for Brillouin-zone integration to reduce the computational cost.

**Figure 1:**
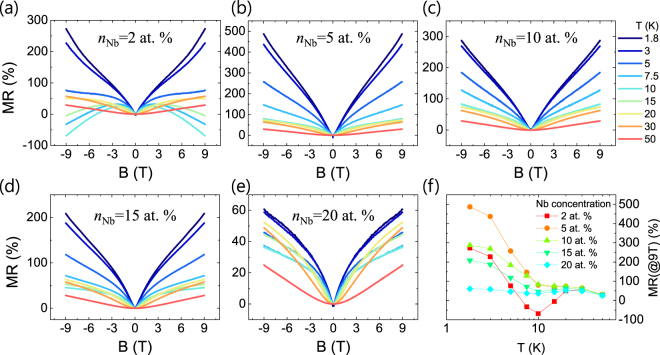
(**a**~**e**) Magnetoresistance (MR) vs. *B* curves of Nb:STO epitaxial films with varying Nb concentration (*n*_Nb_) at various temperatures. The color code of all plots is same as that in (**c**). (**f**) Temperature dependence of MR at 9 T of Nb:STO films.

Figure 1(a)~(e) show the magnetoresistance ($MR(B)=\frac{R(B)-R(0)}{R(0)}\times 100$ (%)) of Nb:STO films (*n*_Nb_ = 2, 5, 10, 15, 20 at. %) as a function of the applied magnetic field (*B*) at various temperature (*T*), where *B* is applied perpendicular to the film surface. A few intriguing features can be found. First, at low temperature (*T* < 20 K), the Nb:STO films show a sharp and linear MR vs. *B* curve at low field region (|*B*| < ~2 T) independent of *n*_Nb_. This behavior is consistent with the LMR shown in heavily-doped Sr(Nb_0.2_Ti_0.8_)O_3_ epitaxial films presented in our previous report^17^. Second, a lightly-doped Sr(Nb_0.02_Ti_0.98_)O_3_ film has a peculiar *T*-dependence of the MR vs. *B* curve. In detail, in the temperature range from 7.5 to 15 K, MR decreases with increasing the strength of *B* in the high field region (|*B*| > 2 T) leading to a high negative MR of about −70 % at 10 K and 9 T. In contrast, the other samples do not show such a negative MR although they all show an anomalous behavior (minimum MR or change of slope) around 10 K as shown in Fig. 1(f). Similar behaviors, including the LMR, were observed in top-gated LaAlO_3_/SrTiO_3_ devices, where the sign change of MR was induced by electric field^12,24^. The authors explained those phenomena in terms of the change of the strength of WAL effect resulting from the change in the Rashba SOC constant by the top-gate bias^25^. The strength of WAL effect also depends on *T*, which is given by the sum of spin-orbit, spin-flip, and inelastic scatterings^18,19^. Therefore, the observed complex *T*-dependence of the MR vs. *B* curve can be associated with *T*-dependence of the WAL effect. In contrast to LaAlO_3_/SrTiO_3_ where the conduction is confined in the atomically-thin two dimensional (2D) channel, Nb:STO films are assumed to have three dimensional (3D) transport because their thickness is ~16 nm comparable to the mean free path. The 3D WAL effect is described by Fukuyama-Hoshino (F-H) model^26–28^, where MR is given by the following equations (1) and (2).

1 $$\frac{\Delta {\rho }_{WAL}(B)}{\rho {(0)}^{2}}=\frac{{e}^{2}}{2{\pi }^{2}h}\sqrt{\frac{2\pi eB}{h}}[\frac{1}{2}{f}_{3}(\frac{B}{{B}_{\varphi }})-\frac{3}{2}{f}_{3}(\frac{B}{{B}_{2}})]$$

2 $${f}_{3}(y)=\sum _{n=0}^{\infty }[2{(n+1+\frac{1}{y})}^{\frac{1}{2}}-2{(n+\frac{1}{y})}^{\frac{1}{2}}-{(n+\frac{1}{2}+\frac{1}{y})}^{-\frac{1}{2}}]$$

In Eq. (1), *e* and *h* are the charge of an electron and Planck’s constant, respectively. And, ${B}_{\varphi }={B}_{i}+2{B}_{S}$ and ${B}_{2}={B}_{i}+\frac{2}{3}{B}_{S}+\frac{4}{3}{B}_{SO}$, where ${B}_{x}=\frac{\hslash }{4eD{\tau }_{x}}$ and *τ*_*x*_s (where *x* = *i, S*, and *SO*) are the characteristic time for the inelastic, spin-flip, and spin-orbit scattering, respectively. And, $\hslash$ and *D* are the reduced Planck’s constant (=*h*/2π) and diffusion coefficient of electron in Nb:STO, respectively.

**Figure 2:**
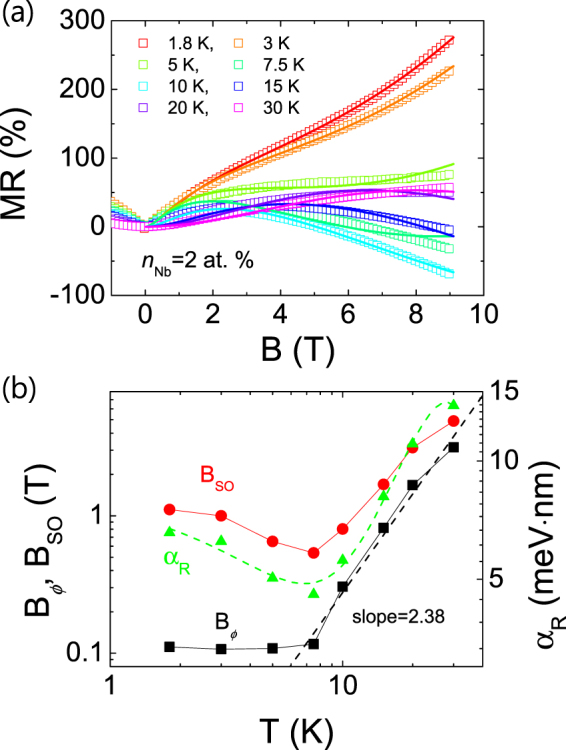
(**a**) MR vs. *B* curves of a Nb:STO film (*n*_Nb_ = 2 at. %) measured at various temperatures (symbol) together with fitting curves obtained by Eq. (3) (solid lines, see text). (**b**) Temperature dependence of the phase-coherence field (*B*_*ϕ*_, black solid square), the spin-orbit field (*B*_SO_, red solid circle), and the Rashba SOC constant (*α*_R_, green solid triangle). A black dashed line is a linear fit to *B*_*ϕ*_(*T*) curve for *T* above 7.5 K in the log-log scale, showing a power-law dependence of *B*_*ϕ*_(*T*). A green dashed line is a fitting curve to the experimental *α*_R_(*T*) assuming a quadratic dependence of Landé g-factor (see text).

In Fig. 2(a), *T*-dependent MR vs. *B* curves of a Nb:STO film (*n*_Nb_ = 2 at. %) are replotted along with the respective fitting curves, which are given by the following equation (Eq. (3))^29,30^.

3 $$MR(B,T)=C\ast R(0,T)\ast \frac{{e}^{2}}{2{\pi }^{2}h}\sqrt{\frac{2\pi eB}{h}}[\frac{1}{2}{f}_{3}(\frac{B}{{B}_{\varphi }(T)})-\frac{3}{2}{f}_{3}(\frac{B}{{B}_{2}(T)})]+k{(\frac{B}{R(0,T)})}^{2}$$

The first and the second term originate from the WAL and the classical orbital motion, respectively, where the latter is assumed to follow the Kohler’s rule^31^ with *k* being a constant independent of *T*. *C* is a constant which depends only on the geometry of the device and *R*(0, *T*) is the resistance under zero magnetic field at various *T*. Consequently, two fitting parameters, *B*_*ϕ*_ and *B*_2_, are used except for the curve at 1.8 K, at which *k* is also used as a fitting parameter and kept constant for the other temperatures. As shown in Fig. 2(a), it is found that Eq. (3) well reproduces the measured MR(*B*) curves in the investigated temperature range (1.8~30 K) even though the observed *T*-dependence is rather complex. The obtained values of *B*_*ϕ*_ and *B*_SO_ are plotted as a function of *T* in Fig. 2(b), which are in the similar range to those of top-gated LaAlO_3_/SrTiO_3_ devices, 0.1 T < *B*_SO_ < 10 T depending on the top-gate bias and 0.05 T < *B*_*ϕ*_ < 0.4 T at 1.5 K^12,24^. Above 7.5 K, *B*_*ϕ*_ increases with *T* faster than *B*_SO_ indicating that the inelastic scattering by phonon is as important as SOC above ~15 K. *T*-dependence of *B*_*ϕ*_ is shown to follow a power-law behavior (*B*_*ϕ*_~*T*^ *p*^) for *T* > 7.5 K as shown in the linear fit in Fig. 2(b). According to ref.^32^, *p* is expected to be 3/2 for electron-electron (*e*-*e*) scattering, 2 for inelastic scattering by transvers*e* phonon, and 3 for inelastic scattering for longitudinal phonon, respectively. In Fig. 2(b), *p* is estimated to be ~2.38 indicating that, above 7.5 K, the phase relaxation is dominated by the phonon scattering with mixed contributions from the transverse and longitudinal phonons.

Below 7.5 K, *B*_SO_ is much higher than *B*_*ϕ*_, indicating that SOC dominates the scattering in that temperature range. An intriguing feature in Fig. 2(b) is the appearance of a minimum *B*_SO_ at 7.5 K, which reminds of the aforementioned anomalous behavior of MR around 10 K presented in Fig. 1(f). Therefore, it seems to imply that the *T*-dependence of *B*_SO_ is closely related to the “10 K anomaly” of MR.

As a possible scenario to explain the *T-*dependence of *B*_SO_, we consider the *T*-dependent Rashba constant (*α*_R_) originating from the *T*-dependence of Landé *g*-factor (*g*). In detail, assuming that the spin relaxation is dominated by the D’Yakonov-Perel (D-P) mechanism, *B*_SO_ can be expressed in terms of *α*_R_ by ${B}_{SO}=\frac{{\alpha }_{R}^{2}{m}^{\ast 2}}{e{\hslash }^{3}}$, where m* is the effective mass of an electron^27,33^. Figure 2(b) also shows the calculated *α*_R_(*T*) (green triangle symbol) using the above relation and the known value of m*(=7.5*m*_0_, *m*_0_ = the rest mass of an electron) in Nb:STO, as m* = (7.3~7.7)*m*_0_ was previously reported^34^. In addition, neglecting the effect of the band structure on SOC, *α*_R_ is expressed in terms of *g* as ${\alpha }_{R}=g(1-g)\frac{\pi e{\hslash }^{2}\varepsilon }{4{m}^{2}{c}^{2}}$, where *ε* and *c* are the electric field generated by the asymmetric structure and the speed of light, respectively^35^. In previous studies^36–39^, it has been shown that *g* depends on *T*, implying that both *α*_R_ and *B*_SO_ should also depend on *T*. With the specific form of *g*(*T*) depending on the material, the observed *α*_R_(*T*) is well reproduced by an assumption of a quadratic *T*-dependence of *g* (*g*(*T*) = *g*_0_ + *g*_1_*T* + *g*^2^*T* ^2^) similar to CdTe case^36^, as shown by the fitting curve (green dashed line in Fig. 2(b)). It implies that the anomalous behavior of MR(*T*) and *α*_R_(*T*) is associated with the *T*-dependence of *g* although the specific form of *g*(*T*) of Nb:STO needs to be measured for confirmation. As another possibility, it might be associated with the unknown phase of heavily-doped Nb:STO epitaxial films under strain. In our previous work, it was found that a heavily doped Nb:STO film (*n*_Nb_ = 20 at. %) showed an upturn in resistivity and magnetic moment below 10 K as well as a local minimum in Hall carrier density around 10 K^17^. Further studies are needed to clarify the origin of the phenomenon.

**Figure 3:**
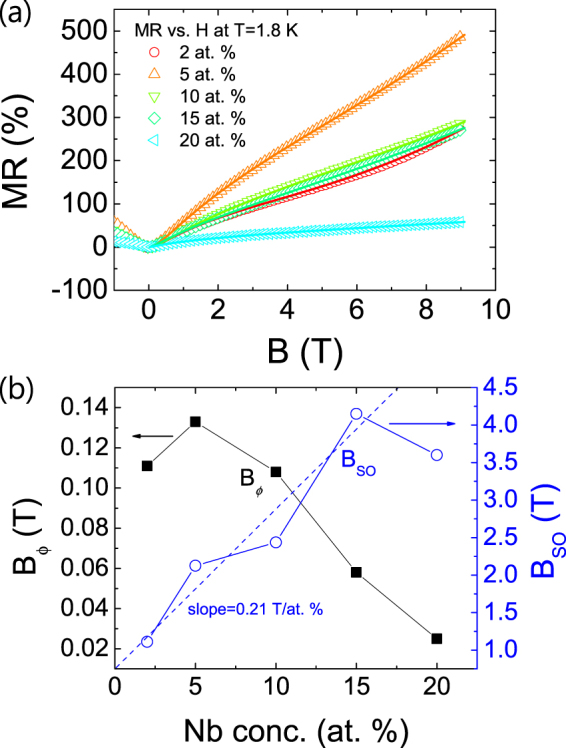
(**a**) MR vs. *B* curves of a Nb:STO films with varying *n*_Nb_ measured at 1.8 K (symbol) together with fitting curves obtained by Eq. (3) (solid lines, see text). (**b**) *B*_*ϕ*_ (black solid square) and *B*_SO_ (blue open circle) as a function of *n*_Nb_.

With a plausible explanation of the MR vs. *B* behavior using the above model (Eq. (3)), MR vs. *B* curves of Nb:STO films with varying *n*_Nb_ at 1.8 K are plotted in Fig. 3(a) to investigate the *n*_Nb_-dependence of SOC. The respective fitting curves are also plotted, providing the information of the dependence of *B*_*ϕ*_ and *B*_SO_ on *n*_Nb_ as shown in Fig. 3(b). For all doping concentrations, it is found that *B*_SO_ is much higher than *B*_*ϕ*_ indicating that SOC is the dominant scattering mechanism in Nb:STO epitaxial films in the low temperature region. Except at *n*_Nb_ = 20 at. %, it is found that *B*_SO_ linearly increases with *n*_Nb_ consistent with an expectation, since SOC is expected to be higher for a compound composed of elements with the higher atomic number (*Z*) elements (*Z*_Nb_ = 41 compared to *Z*_Ti_ = 22). As for the origin of a decrease in *B*_SO_ and a drastic reduction in MR at *n*_Nb_ = 20 at. %, we suggest that a new band might participate in the carrier transport as the Fermi level (*E*_F_) increases over a threshold. A few evidences supporting this explanation are presented in the following.

**Figure 4:**
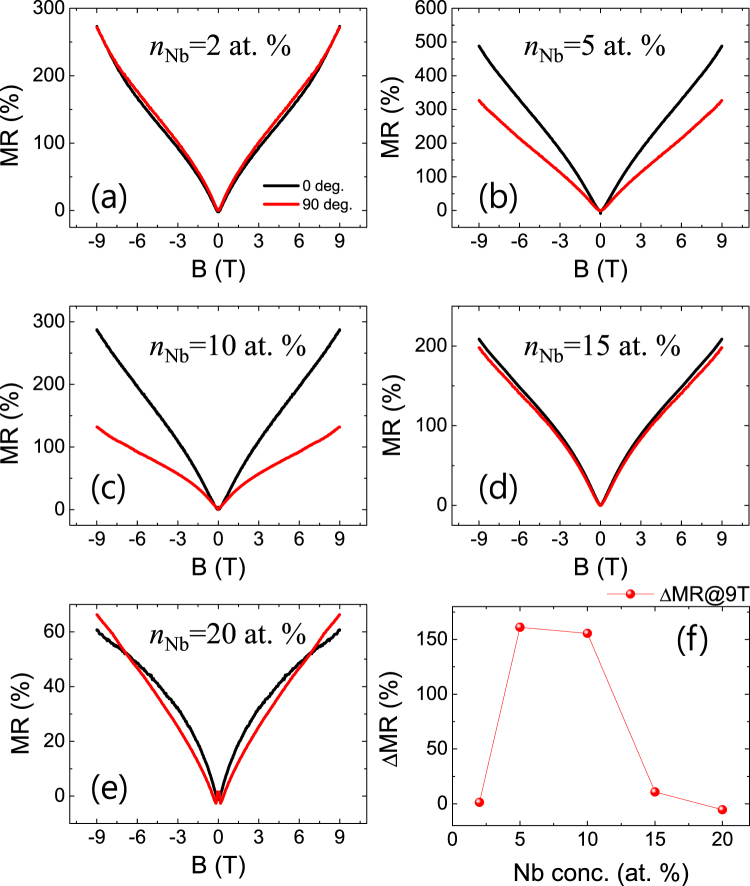
(**a**~**e**) MR vs. *B* curves of Nb:STO epitaxial films at 1.8 K under the out-of-plane (0 deg., black) and the in-plane (90 deg., red) magnetic field. (**f**) Difference in MR (*Δ*MR = MR_perp_-MR_para_) at 9 T as a function of *n*_Nb_.

**Figure 5:**
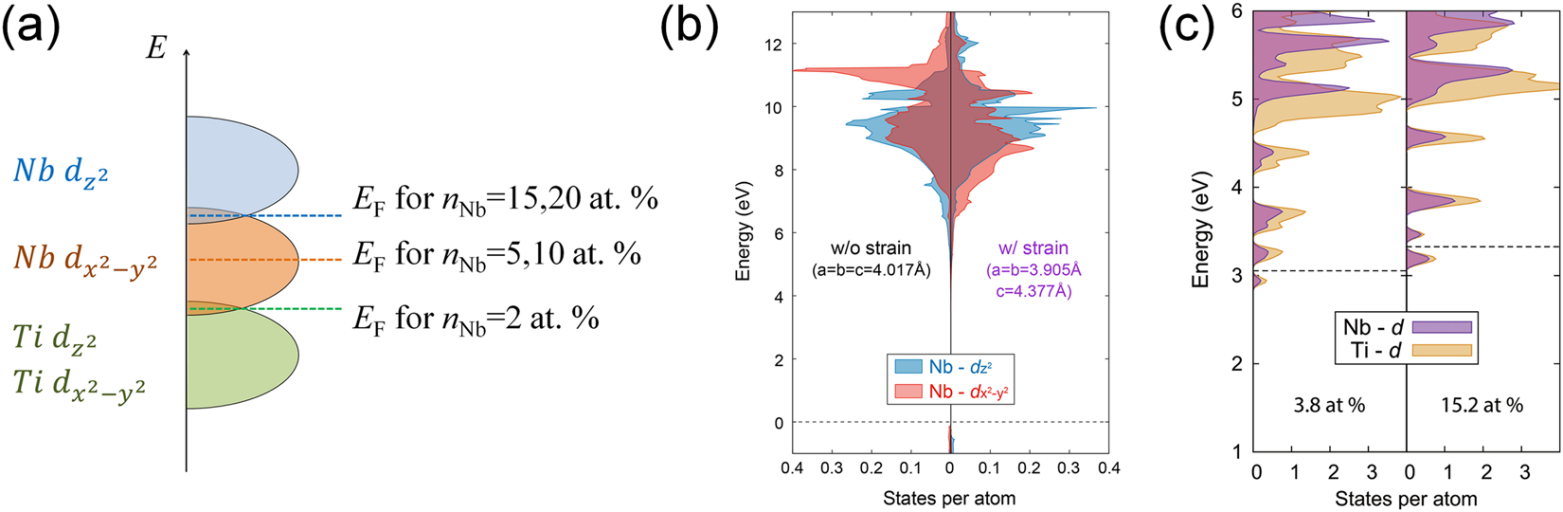
(**a**) A schematic illustration of the orbital-resolved band structure of Nb-doped SrTiO_3_ epitaxial film grown on SrTiO_3_. (**b**) Orbital-resolved band structure of unstrained SrNbO_3_ film (left panel, *a* = 4.017 Å) and strained SrNbO_3_ film with the in-plane lattice constant same as SrTiO_3_ (right panel, *a* = 3.905 Å). Dashed-line shows the position of valence band maximum. (**c**) Orbital-resolved band structure of Sr(Nb_x_Ti_1−x_)O_3_ for x = 3.8 at % and 15.2 at %. The *E*_F_ of each model is shown as dashed lines.

In Fig. 4(a)~(e), MR vs. *B* curves of Nb:STO films at 1.8 K are presented with *B* oriented out-of-plane (MR_perp_, 0 deg.) and in-plane (MR_para._,90 deg.), respectively. For the in-plane MR, the current is still applied perpendicular to *B*. The difference of MR (*Δ*MR = MR_perp_-MR_para_ at 9 T) is plotted as a function of *n*_Nb_ in Fig. 4(f). It is interesting to note that a large anisotropy of MR is found for *n*_Nb_ = 5 at. % and *n*_Nb_ = 10 at. % samples whereas a negligibly small anisotropy is observed for the others. This observation seems to imply a change in the shape of the conduction band depending on *n*_Nb_. The band structure of *n*_Nb_ = 2, 15, and 20 at. % samples should be symmetric along *k*_*x*_ (or *k*_*y*_) and *k*_*z*_ direction in *k*-space while that of *n*_Nb_ = 5 and 10 at. % samples is expected to be asymmetric. This change in the shape of the conduction band depending on *n*_Nb_ might be associated with the crystal field splitting of Nb *d*-orbitals. It was known that the conduction band of Nb:STO is composed of degenerate Ti *d*-orbitals and Nb *d*-orbitals^40–42^. Since the Nb:STO film epitaxially grown on STO is strained as shown in XRD data (see Figure S1 in the Supplementary Information), *d*-orbitals of Nb atom are exerted by the crystal field possibly resulting in the splitting of energy levels. Consequently, the orbital-resolved conduction band structure of a Nb:STO film is expected to be similar to that seen in Fig. 5(a). For low *n*_Nb_, if *E*_F_ is located within the overlap between degenerate Ti-*d* bands and Nb-${d}_{{x}^{2}-{y}^{2}}$ band, the transport should be symmetric along *k*_*x*_ (or *k*_*y*_) and *k*_*z*_ direction. Since Nb substituting Ti produces electron carriers^43,44^, *E*_F_ increases with *n*_Nb_. As *n*_Nb_ increases, *E*_F_ enters the region where the conduction band is contributed mainly from Nb-${d}_{{x}^{2}-{y}^{2}}$ band resulting in 2D transport nature. As *E*_F_ increases further, Nb-${d}_{{z}^{2}}$ starts to contribute to the conduction band again making the conduction along *k*_*z*_ direction significant. This picture can naturally explain the change of the MR anisotropy depending o*n n*_Nb_.

To verify the assumed band structure, we have performed the orbital-resolved band calculation using density functional theory (DFT), which is shown in Fig. 5(b). For simplicity, we have calculated the band structure of SrNbO_3_, which can provide qualitative but essential information on the change of the band structure on doping Nb in SrTiO_3_. The left and right panels show the partial density of states (PDOS) for an unstrained SrNbO_3_ (*a* = 4.017 Å)^22,45^ and a strained film epitaxially grown on SrTiO_3_ (*a* = 3.905 Å)^23^, respectively. Comparing with the unstrained SrNbO_3_, it is found that Nb-${d}_{{x}^{2}-{y}^{2}}$ band moves to lower energy whereas Nb-${d}_{{z}^{2}}$ band moves to higher energy consistent with our expectation described above. To compare the relative position of Nb-*d* in Nb:STO, we also calculate the PDOS of Nb:STO (*n*_Nb_ = 3.8 at. % and 15.2 at %) as shown in Fig. 5(c). It is shown that the position of Nb-*d* composing the conduction band is right above the position of Ti-*d* as we assumed in Fig. 5(a). Comparing results of differe*n*t *n*_Nb_, it is also shown that *E*_F_ increases with *n*_Nb_. Therefore, we believe that the crystal-field splitting of the Nb *d*-bands in Nb:STO films is associated with the observed change in the anisotropy of MR. As alternative explanation, the interaction of *d*-electrons under a strong SOC should be addressed because it was reported to be important in material systems including transition metal elements^46^.

In summary, we have investigated magnetotransport properties of epitaxial Nb:STO thin films to find the effect of Nb on the SOC strength. The magnetotransport properties are well described by the F-H model describing 3D WAL and the estimated SOC strength is found to be proportional to *n*_Nb_. Furthermore, a few intriguing phenomena are presented, non-monotonic temperature dependence of the SOC strength and anisotropy of MR in the intermediate *n*_Nb_ region, which might be explained in terms of the temperature dependence of the *g*-factor and the change in band structure with *n*_Nb_. This result demonstrates that the epitaxial Nb:STO film basically possesses the strong SOC, whose strength can be controlled by *n*_Nb_. We believe that these results not only provide a material platform to study the interplay between SOC and strong correlation, which is an interesting subject in condensed-matter physics, but also enable us to design advanced spintronic devices and other novel functional devices combining the strong SOC and other versatile functionalities of strongly-correlated oxides.

## Electronic supplementary material

Supplementary Information
