# Supplementary material for: Effect of Nb concentration on the spin-orbit coupling strength in Nb-doped SrTiO3 epitaxial thin films
Source: Sci Rep. 2018 Apr 10;8:5739. doi: 10.1038/s41598-018-23967-2 (PMC5893625; doi:10.1038/s41598-018-23967-2)
Supplement: Supplementary file 1 — Supplementary Information [file 41598_2018_23967_MOESM1_ESM.pdf]

Supplementary Information for

Effect of Nb-concentration on the spin-orbit coupling strength in Nb-doped $SrTiO_3$ epitaxial thin films

Seong Won Cho[1, 2], Milim Lee[1], Sungmin Woo[3], Kanghoon Yim[4], Seungwu Han[4], Woo Seok Choi[3], and Suyoun Lee[1,2*]

S1. Thin film growth and structural characterization

Nb:STO concentration split films were fabricated at 700 °C in 10^{-5} Torr of oxygen partial pressure using pulsed laser epitaxy (PLE). Laser (248 nm; IPEX 864, Lightmachinery, Nepean, Canada) fluence of 1.5 J/cm^2 and repetition rate of 5 Hz was used. $SrTiO_3$ and $Sr(Nb_{0.2}Ti_{0.8})O_3$ targets were used to systematically modify the Nb:STO concentration, by controlling the ablation ratio between the two targets within a unit cell thickness. The advantage of using co-ablation of two targets instead of using Nb:STO solid solution target with different n_{Nb} was to avoid additional complexity and quality issues due to preparing distinctive targets. The thickness of the Nb:STO thin films was 16 ± 1 nm, as measured by X-ray reflectometry (XRR). The atomic structure and epitaxy relation of the thin films were characterized using high-resolution X-ray diffraction (XRD) (Rigaku, Smartlab).

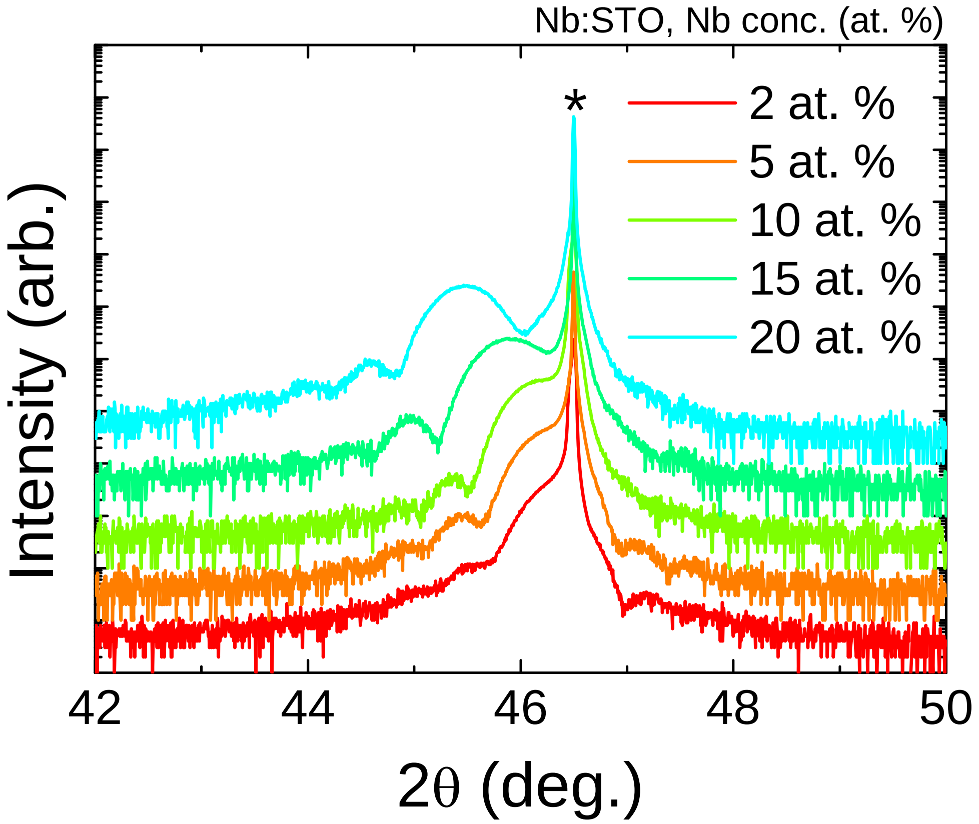

Figure S1. XRD θ–2θ scans for $Sr(Nb_xTi_{1-x})O_3$ thin films on $SrTiO_3$ substrates.
